# Persistent High Leptospiral Shedding by Asymptomatic Dogs in Endemic Areas Triggers a Serious Public Health Concern

**DOI:** 10.3390/ani11040937

**Published:** 2021-03-26

**Authors:** Ricardo Sant’Anna da Costa, Maria Isabel N. Di Azevedo, Ana Luiza dos Santos Baptista Borges, Filipe Anibal Carvalho-Costa, Gabriel Martins, Walter Lilenbaum

**Affiliations:** 1 Laboratório de Bacteriologia Veterinária, Departamento de Microbiologia e Parasitologia, Universidade Federal Fluminense, Niterói, 24210-130 Rio de Janeiro, Brazil; ricardo-santanna@hotmail.com (R.S.d.C.); isabeldiazevedo@gmail.com (M.I.N.D.A.); analuizaborges@id.uff.br (A.L.d.S.B.B.); gmartins@id.uff.br (G.M.); 2 Laboratório de Epidemiologia e Sistemática Molecular, Instituto Oswaldo Cruz (Fiocruz), 21040-900 Rio de Janeiro, Brazil; carvalhocosta70@hotmail.com

**Keywords:** canine leptospirosis, silent leptospiral infection, subclinical infection

## Abstract

**Simple Summary:**

Dogs are known as hosts of *Leptospira interrogans* and can spread this bacterium to the environment. Although Canicola is responsible for determining chronic disease in dogs, when affected by incidental serogroups such as Icterohaemorrhagiae, acute disease may occur with a predominance of clinical signs with hepatic and renal lesions. In endemic areas, it is a serious public health problem, as dogs can become asymptomatic carriers of leptospires in the urine, characterizing a risk in the context of zoonotic leptospirosis. Thus, this study aims to estimate the incidence and duration of the elimination of leptospires in the urine of dogs, taking another step from a previous study of our group, by a longitudinal, long-term and molecular approach. A total of 125 dogs without signs of leptospirosis were included in the study. Of the 125 dogs, 62 were PCR-positive (48.8% (95% CI, 47.9–49.7%)) throughout the study. Most dogs were shedding the Icterohaemorrhagiae serogroup in urine, which was unexpected, since the animals remained clinically asymptomatic during the study. Although the fact that asymptomatic dogs eliminate leptospires is not new, the extent of this fact and that the strain is virulent is impressive, with an impact on public health that cannot be overlooked.

**Abstract:**

(1) Background: Dogs are known as hosts of *Leptospira interrogans* and can spread this bacterium to the environment. Although Canicola is responsible for determining chronic disease in dogs, when affected by incidental serogroups such as Icterohaemorrhagiae, acute disease may occur with a predominance of clinical signs with hepatic and renal changes. In endemic areas, it is a serious public health problem. Thus, this study aims to estimate the incidence and duration of elimination of leptospires in the urine of dogs, taking another step from a previous study of our group, by a longitudinal, long-term and molecular approach. (2) Methods: A total of 125 dogs without apparent symptoms of leptospirosis were included in the study. The dogs were all PCR-negative and seronegative at the beginning of the study. Blood samples were collected for hematological examinations and urine for amplification of the lipL32 gene by PCR at five different time points during one year. (3) Results: Out of the 125 dogs, 62 became lipL32 PCR-positive (48.8% (95% CI, 47.9–49.7%)) at some point during the study, distributed as follows: at day 0, all negative; day 90, 18/125 (14.4% (95% CI, 13.5–15.3%)); day 180, 18/125 (14.4% (95% CI, 13.5–15.3%)); day 270, 12/125 (9.6% (95% CI, 8.7–10.5%)); and day 365, 14/125 (11.2% (95% CI, 10.3–12.1%)). Out of the 62 amplicons, 22 were sequenced, targeting a short region of secY gene. Of these, 20 (90.9%) were identical to the *L. interrogans* serovar Icterohaemorrhagiae, while two (9.1%) were *Leptospira noguchii*. (4) Conclusions: The fact that the leptospires of the Icterohaemorrhagiae serogroup were characterized was unexpected, since the animals remained clinically asymptomatic during the study. The fact that asymptomatic dogs shed leptospires is not new, but the extent of this fact and the characterized strain is impressive, with an impact on public health that cannot be overlooked.

## 1. Introduction

Leptospirosis is an infectious disease of worldwide distribution, caused by pathogenic species of the genus *Leptospira*. It is characterized by being the most common zoonosis, which is responsible for more than 1 million cases worldwide, with 60,000 deaths per year [1]. It affects domestic and wild animals, including mammals, amphibians, birds, reptiles and possibly fish, which can carry pathogenic species of leptospires. Dogs are known as hosts of the *Leptospira interrogans* serogroup Canicola and can spread this serovar to the environment [2,3]. In addition, dogs are highly susceptible to infection and act as a sentinel species for environmental risk to humans, due to their high level of environmental exposure to pathogenic leptospires [4,5].

Canine leptospirosis is widely described worldwide [4,6]. Leptospires affect the kidneys and are maintained by carrier animals, which shed the bacteria in the urine, becoming a source of infection for humans and other hosts [7,8,9]. Humans are infected by direct or indirect contact with the urine of infected animals, and by contact with contaminated water and/or soil [6]. The role of dogs as carriers has been increasingly studied, as it is known that they can act as a source of infection and, therefore, represent a public health problem [10]. Few studies have been carried out with asymptomatic dogs in endemic regions. A study carried out in Chile indicated 19.3% of dogs shed leptospires [11], and, in Brazil, 20% of dogs shed leptospires in the urine [12]. Nevertheless, it is known that shedding is intermittent, which means that even infected dogs may present negative PCR results. In both studies, urinary samples were collected only once from each dog, which represents a possible bias. Therefore, the number of infected animals, although high, may be underestimated, and the problem may be even bigger. Dogs are pets and are often close to humans, living inside the house and being members of the family. In this context, it is noteworthy that asymptomatic carrier dogs are particularly important for zoonotic transmission as well as for the control of canine leptospirosis, since they remain undiagnosed and untreated, consequently shedding leptospires for long periods.

Considering this, this study aims to estimate the incidence and duration of the elimination of leptospires in the urine of dogs, taking another step from a previous study of our group [12], by a longitudinal, long-term and molecular approach.

## 2. Materials and Methods

### 2.1. Ethics Approval

The study was conducted after approval by the Ethics Committee of Universidade Federal Fluminense (CEUA no 3778190419).

### 2.2. Dogs

A total 125 dogs, no matter the sex, aged 2–9 years were studied, with no apparent symptoms of leptospirosis or any other infectious disease. The dogs were all from a shelter located in the same endemic region of our previous study, the municipality of São Gonçalo, Rio de Janeiro State [12]. All animals were shown to be healthy in clinical inspections, always performed by the same veterinarian. They presented regular hematological exams (complete blood count, biochemical measurements such as serum ALT (alanine aminotransferase), AST (aspartate aminotransferase) activity, urea and creatinine levels) and were unvaccinated for leptospirosis. As an inclusion criterion, only dogs that were seronegative Microscopic Agglutination Test (MAT) and PCR-negative at day 0 were included in the study. Dogs that presented serologically and/or PCR-positive at day 0 were treated with antibiotics and excluded from the study, being maintained in a different area of the shelter. The antigens used at MAT were *Leptospira interrogans* serovars Autumnalis (Akiyami A), Bratislava (Jez-Bratislava), Bataviae (Van Tienen), Canicola (Hond Utrecht IV), Grippotyphosa (Moska V), Icterohaemorrhagiae (RGA), Copenhageni (M 20) and Pomona (Pomona).

### 2.3. The Shelter

The shelter was located in a private urban area, surrounded and divided into covered pens or kennels, and the center of the enclosure was without a covered area. All dogs were mixed, having free access to the kennels and a common area for all. The shelter had a very poor infrastructure, without painting and featuring unfinished work, but was always clean. Rodent control was performed every six months. The entire floor was cemented, and the animals were separated individually or in groups of up to three animals in the pens, but all lived together. Dogs were fed once a day. The shelter received regular visits and assistance from a veterinarian, as well as health surveillance.

### 2.4. Study Design

All the dogs were followed up and evaluated for 365 days. Sampling occurred at days 0, 90, 180, 270 and 365. At each collection, visits were always made by the same veterinarian for a complete clinical examination of the body condition, including variables such as temperature, hydration, mucosal color, tissue perfusion and cardiorespiratory sounds. In addition, monthly blood tests were carried out, whereupon five mL of blood was collected from the animals selected in a tube with and without an ethylenediamino tetra-acetic acid (EDTA) anticoagulant. With the blood samples, a complete blood count was performed (Humacount 60 Ts Produtos para Laboratórios Ltd., an automatic hematology analyzer), as were biochemical tests (Bio-Plus^®^ Biochemical Analyzer, Bioplus Products for Laboratórios Ltda, Cep: 06407-000 Barueri, Sao Paulo, Brazil). In these tests, the serum activity of alanine amino transferase (ALT), aspartate aminotransferase (AST) and alkaline phosphatase was analyzed, in addition to serum dosages of urea and creatinine, using commercial kits (Labtest kits, Vista Alegre, Lagoa Santa, Minas Gerais, Cep 33.240-152, Brazil) according to the manufacturer’s instructions. Urine (for PCR) was also collected from all animals monthly. Urine was collected by ultrasound-guided cystocentesis. Prevalences were calculated using an error of 5% and 95% confidence intervals (CIs) with OpenEpi.

### 2.5. Molecular Analyses

Urine samples were centrifugated to increase the chance of recovering leptospires. DNA from urine samples was extracted by the commercial Wizard SV Genomic DNA Purification System kit following the manufacturer’s instructions (Promega, Madison, WI, USA). PCR assay targeted the lipL32 gene (present majority in pathogenic leptospires) using the primers LipL32-45F (5′-AAG CAT TAC CGC TTG TGG TG-3′) and LipL32-286R (5′-GAA CTC CCA CAG CGA TT-3) in a final volume of 25 µL following conditions described in [13] (p. 7). For each sample set, ultrapure water was used as a negative control, while 10 fg of DNA extracted from *Leptospira interrogans* serotype Copenhageni, strain Fiocruz L1-130, was used as a positive control. All reactions occurred in the Gene Amp PCR System 9700 thermal cycler (Applied Biosystems, Foster City, CA, USA). The total volume of each sample was analyzed by agarose gel electrophoresis (2%), stained with gel red and the DNA bands were visualized under ultraviolet light. The expected size of the amplicon was approximately 240 bp, varying slightly between the different species of *Leptospira* [13].

To confirm the identity of the amplicons, PCR products were selected and subjected to nucleotide sequencing based on the secY gene, after nested PCR following conditions described in [14]. Amplicons were purified with the GFX PCR DNA Gel Band Purification kit (GE HealthCare, Chicago, IL, USA). The nucleotide sequencing reaction was performed using the BigDye Terminator v3.1 kit (Applied Biosystems) and capillary electrophoresis was performed in both directions with an ABI 3730 automatic sequencer (Applied Biosystems). Regarding phylogenetic analysis, Pairwise/Blast/NCBI, SeqMan v. 7.0, ClustalW v. 1.35 [15], and BioEdit v. 7.0.1 [16] softwares were used for editing and sequence analysis. A maximum likelihood (ML) tree was constructed using the K2P model with the gamma distribution in MEGA X software, as this was determined to be the best-fitting model of DNA substitution using the Bayesian information criterion.

## 3. Results

No clinical or hematological/biochemical alterations were observed throughout the study. Out of the 125 dogs, 62 became lipL32-PCR positive (48.8% (95% CI, 47.9–49.7%)) at some point in the study, distributed as follows: at day 0, all negative; day 90, 18/125 (14.4% (95% CI, 13.5–15.3%)); day 180, 18/125 (14.4% (95% CI, 13.5–15.3%)); day 270, 12/125 (9.6% (95% CI, 8.7–10.5%)); and day 365, 14/125 (11.2% (95% CI, 10.3–12.1%)). Eight dogs presented positive more than once, but not consecutively. Of the 62 obtained amplicons, 22 yielded good sequences of the secY gene. Pairwise/Blast/NCBI comparisons with the GenBank secY gene dataset identified 20/22 of them as *L. interrogans* serovar Icterohaemorrhagiae (99.2–100% identity) and 2/22 as *Leptospira noguchii* (both 99.7% identity). The samples identified in the sequencing as *L. interrogans* were found on day 365 (*n* = 7), day 270 (*n* = 3), day 180 (*n* = 6) and day 90 (*n* = 4). Regarding *L. noguchii*, one sample was found on day 365 and the other on day 270.

**Figure 1:**
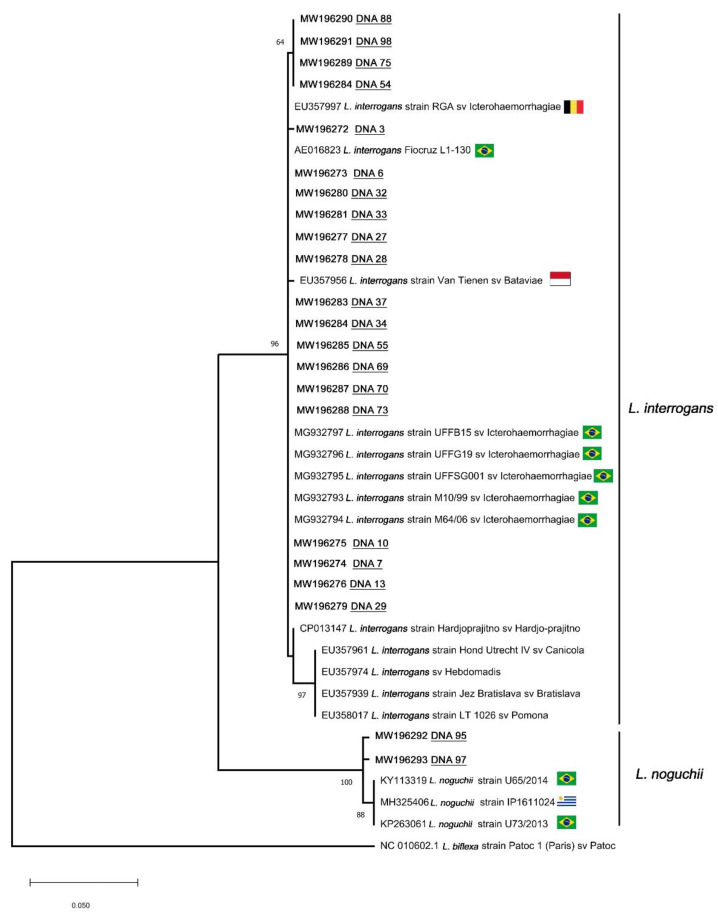
Maximum likelihood phylogenetic tree inferred from partial secY gene sequences of *L. interrogans* and *L. noguchii* from this study (DNA + number) and GenBank sequences (accession numbers, strain and serovar are shown). Geographical localization of sequences is represented by flags. Numbers at nodes are bootstrap values greater than 50%. *Leptospira biflexa* strain Patoc is the outgroup taxa.

Phylogenetic analysis based on ML K2P + G tree confirmed *L. interrogans* and *L. noguchii* species identification with high support values (96% and 100%, respectively). Moreover, *L. interrogans* sequences from the present study clustered together with strains from the serovar Icterohaemorrhagiae, most of them from the same Brazilian geographical region. Similarly, *L. noguchii* species from the present study grouped with sequences from South America. The nucleotide sequences were deposited in GenBank under accession numbers *L. interrogans*: MW196272–MW196291, *L. noguchii*: MW196292–MW196293 (Figure 1).

## 4. Discussion

As described by our group, PCR had already demonstrated a high number of shedders of *Leptospira* in urine; 20% of dogs in a single sample [12]. At that occasion, that rate was considered extremely high and alarming. Similar studies on the prevalence of elimination have been carried out in non-endemic regions, showing the discrepancy in results between endemic and non-endemic areas. In this case, rates of 8.2% in the USA [17], 8% in Scotland [18], 7.1% in Ireland [19], 7% in Dublin [19], 1.5% in Germany [7] and 0.2% in Switzerland [20] have been reported. This low European prevalence is likely due to vaccine-induced immunity over time, as well as location in non-endemic areas. The contrast of these rates with our results indicates that, in endemic tropical regions, the role of asymptomatic dogs as carriers of leptospires and their impact on public health cannot be overlooked. Besides, source populations differ between studies and the prevalence in shelter dogs is expected to be higher than in owned dogs [21]. Nevertheless, all of these studies had a transversal character, testing a single urine sample of each dog. It is well known that urinary shedding of leptospires is intermittent [4], so there is a bias that cannot be neglected, i.e., the possibility that an infected animal yields false-negative results due to intermittency. Therefore, we conducted the present study with the same population of dogs of the previous study, in the same endemic area, but increasing the duration of the study and the number of collections, aiming to reduce the bias of the results. As a result, we observed an impressive increase in the number of shedders.

The positivity in urinary PCR at any moment of this study in almost half of the dogs represents serious consequences for the spreading canine leptospirosis and a serious risk to public health. It is noteworthy that all animals were exposed to the same environmental conditions and in the same place, and that infection occurred naturally. It is also important to highlight that this study was not carried out with a large and representative population of dogs, but it is proposed to be a cohort with a defined group of animals naturally exposed to leptospires. Although the intentional selection of animals may represent a study bias, the inclusion and exclusion criteria make the population as homogeneous as possible, reducing the impact of selection on the results presented.

Another interesting outcome of the study was the characterization of *Leptospira noguchii* on 9.1% (2/22) of the sequenced amplicons, since there are not many studies showing this leptospiral species in dogs. It has been reported in different hosts, such as wild animals [22] and domestic animals [23], mainly in the Americas. It has also been described as causing chronic diseases in humans [23]. In a study in Nicaragua, *L. noguchii* was found in canines, suggesting a correlation between the infection of domestic animals and the increasing incidence of human cases [24]. It was also reported in a study the occurrence of *L. noguchii* in southern Brazil, where three strains of *L. noguchii* were isolated, including two in humans and one in a dog, leading to severe clinical disease in the humans and the dog’s death [25]. Therefore, the role of these strains on canine infection as well as their impact on public health cannot be neglected, and remain to be elucidated.

The most unexpected outcome of the study was the molecular evidence that these asymptomatic dogs were shedding an Icterohaemorrhagiae strain, most likely L1-130, a highly virulent strain, important in the incidental infections of dogs worldwide [3,26]. It is correct that strain characterization was not possible in all the dogs, but only in those 22 that yielded good sequences. Nevertheless, the high predominance of this strain indicates with some dose of certainty that it was majorly circulating among these dogs. This fact had been suggested in our previous study [12], but at that moment no molecular characterization was performed, only serology of the dogs, which indicated high predominance of the serogroup Icterohaemorrhagiae (92.7% of the seropositive samples). It is noteworthy that the serogroup Icterohaemorrhagiae is composed not only of the serovar Icterohaemorrhagiae, but also of the serovar Copenhageni. The most frequent leptospires related to severe cases in Brazil are Icterohaemorrhagiae and Copenhageni, from the same serogroup [27,28]. This serovar, particularly the strain FIOCRUZ L1-130, is known as the most prevalent strain in human leptospirosis as well as in dogs [29], causing severe acute disease and death. Besides, it has been demonstrated by our group that in Brazil all Icterohaemorrhagiae strains belong to the same clonal complex, independently of being recovered from rats, humans or ill dogs [29]. In the present study, we also include in the group asymptomatic dogs, which spread the same bacterium as the clinically diseased dogs.

Icterohaemorrhagiae strains are known to be highly virulent to dogs and humans, leading to severe clinical disease and very often death. The fact that the dogs of the present study became maintenance hosts of this strain without developing clinical disease was highly unexpected, and the reason why it happened remains to be elucidated, which indicates that the agent–host–environment relationship still requires a lot of research to be fully understood.

## 5. Conclusions

The shedding of leptospires by asymptomatic dogs in endemic areas is probably much more common than expected and has been largely neglected. A high number of naturally exposed animals became shedders during the study, and a highly virulent strain of serogroup Icterohaemorrhagiae was being shed by the majority of the dogs. The impact of these outcomes on public health cannot be neglected.

## Data Availability

Nucleotide sequences from this study can be found at GenBank (https://www.ncbi.nlm.nih.gov/genbank/).
